# A Decision Aid for COPD patients considering inhaled steroid therapy: development and before and after pilot testing

**DOI:** 10.1186/1472-6947-7-12

**Published:** 2007-05-15

**Authors:** Elie A Akl, Brydon JB Grant, Gordon H Guyatt, Victor M Montori, Holger J Schünemann

**Affiliations:** 1 Department of Medicine, University at Buffalo, Buffalo, NY, USA; 2 Department of Social & Preventive Medicine, University at Buffalo, Buffalo, NY, USA; 3 Department of Medicine, VAMC, Buffalo, NY, USA; 4 Department of Biostatistics, University at Buffalo, Buffalo, NY, USA; 5 Department of Physiology and Biophysics, University at Buffalo, Buffalo, NY, USA; 6 Department of Medicine, McMaster University, Hamilton, Ontario, Canada; 7 Department of Clinical Epidemiology and Biostatistics, McMaster University, Hamilton, Ontario, Canada; 8 Knowledge and Encounter Research Unit, Mayo Clinic College of Medicine, Rochester, Minnesota, USA; 9 INFORMA, Dept. of Epidemiology, Italian National Cancer Institute Regina Elena, Rome, Italy

## Abstract

**Background:**

Decision aids (DA) are tools designed to help patients make specific and deliberative choices among disease management options. DAs can improve the quality of decision-making and reduce decisional conflict. An area not covered by a DA is the decision of a patient with chronic obstructive pulmonary disease (COPD) to use inhaled steroids which requires balancing the benefits and downsides of therapy.

**Methods:**

We developed a DA for COPD patients considering inhaled steroid therapy using the Ottawa Decision Support Framework, the best available evidence for using inhaled steroid in COPD and the expected utility model. The development process involved patients, pulmonologists, DA developers and decision making experts. We pilot tested the DA with 8 COPD patients who completed an evaluation questionnaire, a knowledge scale, and a validated decisional conflict scale.

**Results:**

The DA is a computer-based interactive tool incorporating four different decision making models. In the first part, the DA provides information about COPD as a disease, the different treatment options, and the benefits and downsides of using inhaled steroids. In the second part, it coaches the patient in the decision making process through clarifying values and preferences. Patients evaluated 10 out of 13 items of the DA positively and showed significant improvement on both the knowledge scale (p = 0.008) and the decisional conflict scale (p = 0.008).

**Conclusion:**

We have developed a computer-based interactive DA for COPD patients considering inhaled steroids serving as a model for other DAs in COPD, in particular related to inhaled therapies. Future research should assess the DA effectiveness.

## Background

Increasingly, patients want to become actively involved in medical decision-making [1]. Active patient involvement can improve outcomes including quality of life and can possibly reduce health care expenditures [2-4]. However, therapy and screening decisions are complicated for several reasons. First, there is often no single 'best' choice because people vary in the values or personal importance that they place on the benefits and harms of different treatment or screening options. Second, the evidence needed to trade off benefits and downsides (harm, burden and cost) of options may be of low quality. Third, clinicians have little knowledge about the best ways to present evidence and to involve patients in the decision making process. Individual practice circumstances further complicate evidence based decision making.

Because of the existing evidence that active patient involvement can improve outcomes [2-4] and in order to help people make wise choices among options [5,6] investigators and clinicians have developed decision aids (DAs). DAs are decision support tools that provide patients with detailed and specific information on options and outcomes, help them clarify their values, and guide them through the decision making process [7]. DAs are superior to usual care interventions in improving knowledge and realistic expectations of the benefits and harms of options; reducing passivity in decision making; and lowering decisional conflict due to feeling uninformed [7]. Additionally, they assist patients with chronic diseases in feeling better socially supported and potentially improving their behavioral and clinical outcomes [8].

Chronic Obstructive Pulmonary Disease (COPD) is a major cause of chronic morbidity and mortality throughout the world [9]. In the US alone, COPD affected an estimated 11 million adults in 2002, and was the fourth leading cause of death in 2004 [10]. The 2004 US expenditures for health and lost productivity due to COPD were estimated at $37.2 billions [10]. Further increases in COPD prevalence and mortality will occur in the coming decades [11].

Inhaled steroids, one of therapeutic options for COPD, reduce the number of acute exacerbations in COPD patients [12,13] and have a small beneficial effect on their health related quality of life (HRQL), but have a number of side effects including oropharyngeal candidiasis and skin bruising [12]. They also pose the additional burden of using an inhaler. Asking COPD patients to decide about starting inhaled steroids implies a decision making process trying to balance the benefits and harms by including their personal values. Although we did not identify any study about the difficulties with making such a decision, we had noted difficulties in the course of our clinical practice and when speaking to colleagues. In this paper we describe the development and pilot testing of a decision aid for COPD patients considering inhaled steroids therapy that can serve as a model for other decisions aids in COPD.

## Methods

### Development of the Decision Aid

The development of the COPD DA consisted of five steps:

***(1) Structural development***: the structure of the DA follows the Ottawa Decision Support Framework (DSF) [14] and is based on the Ottawa Personal Decision Guide (OPDG) [15]. The framework is an evidence-based, practical, mid-range theory for guiding patients making health or social decisions. Mid-range theories are moderately abstract and inclusive theories that address specific phenomena and are composed of concepts and propositions that are measurable [16]. The framework supports decision making through providing information about the disease, its treatment alternatives and the associated outcomes; through clarifying values; and finally through augmenting skills in decision making. The Ottawa Personal Decision Guide (OPDG) is a generic decision aid designed for any health-related and/or social decision. It helps people assess their decision making needs, plan the next steps, and track their progress in decision making. However, it does not include standardized guidance about how to include an interactive computer based interface, which was one of the aims of our project. Furthermore, opposed to the generic features of the OPDG we aimed to include different decision making models in the aid. The latter was another of our specific aims for this decision aid and the intended decision making process.

***(2) Information compilation***: we derived the information about the outcomes of inhaled steroids treatment in COPD patients from the most recent systematic review of the medical literature about the topic [12], and from several of the original studies included in the review. Beneficial outcomes include a reduction in the rate of exacerbations (RR = 0.70; 95% CI: 0.58 to 0.84: from 0.8 to 0.56 exacerbation per year) [12] and a deceleration in the rate of decline in health status [17]. Harms include increases in the rates of oropharyngeal candidiasis (RR = 2.1; from 0.8% to 1.7% per year), skin bruising (RR = 2.1; from 1.0% to 2.1% per year) and dysphonia (RR = 2.0; from 1.7% to 3.3% per year) as well as the burden of using the drug. We also included a statement about the uncertainty of the effect of inhaled steroids on mortality, cataract and bone fractures [18].

***(3) Platform design***: two professional web designers experienced health related websites developed the platform of the DA using Macromedia Dreamweaver 2002 software. They developed it as a CD-ROM version and then made it available on the World Wide Web.

***(4) Experts' feedback***: we consulted pulmonologists, medical decision making experts (Drs. Amiram Gafni and Cathy Charles, McMaster University, Hamilton, Canada) and a DA expert (Dr. Annette O'Connor, University at Ottawa, Canada). We used their in-depth feedback to improve different aspects of the DA. These improvements related to the structure of the decision aid and the integration of different decision making models.

***(5) Patients' feedback***: we conducted detailed interviews with 7 COPD patients. Each patient reviewed the DA, answered specific questions and provided general comments. After each interview, we made modifications based on the patient feedback. Modifications were mainly related to the use of lay terms, the form of presentation of the statistical presentation, and the amount of information.

### Pilot testing of the Decision Aid

Eight additional COPD patients used the DA as if they were to make a real life decision about the use of inhaled steroids. These participants completed 3 instruments: (1) an evaluation questionnaire; (2) a knowledge scale (before and after use); and (3) a validated decisional conflict scale (before and after use) [19]. The evaluation questionnaire addressed 13 features of the DA that participants rated on 5-point Likert scales (1 for lowest value, 3 for neutral value and 5 for highest value). The knowledge scale consisted of 10 questions we developed specifically about the use of inhaled steroids in COPD patients [see Additional file 1]. We kept track of the required time and the need for assistance in using the DA. The State University of New York at Buffalo and the Buffalo Veterans Affairs Medical Center institutional review boards (IRB) approved this study and all participants provided informed consent.

### Statistical analysis

For the 5 point Likert scale questions, we used a one sample t-test to compare the mean response to 3 (neutral value). For the knowledge and decisional conflict scales, we used a paired t-test to compare the pre and post intervention mean scores. We used SPSS, version 11.0 (SPSS, Inc., Chicago, Illinois) and considered p < 0.01 (two-sided) as statistically significant.

## Results

### The decision Aid [20]

#### Structure

**Figure 1:**
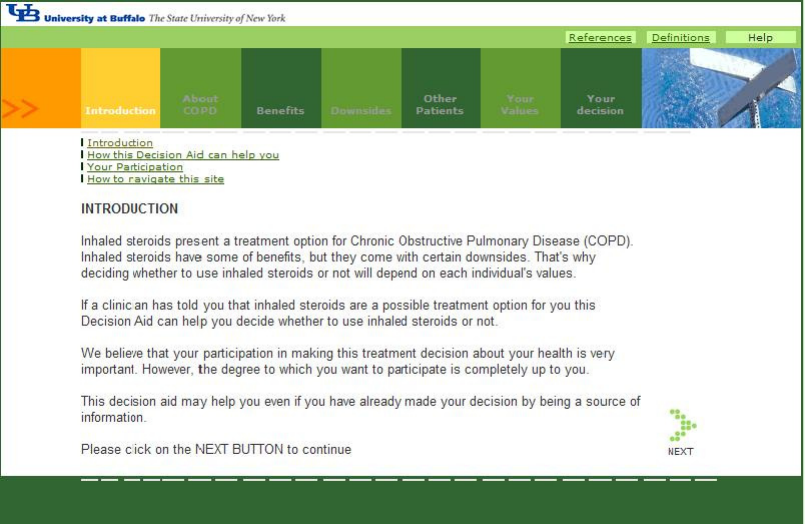
Screenshot showing the general appearance and structure of the DA.

The DA is structured in two parts, each part consists of several sections, and each section consists of several pages (Figure 1) [see Additional file 2]. In the first part, the DA provides medical information about: the use of the DA, COPD, treatment alternatives, the benefits of inhaled steroids, and their downsides. In the second part, the DA coaches the patient in the decision making process through: case scenarios of hypothetical COPD patients using their values to make tradeoffs between the benefits and harms of the inhaled steroids, clarification of the patient's own values for each benefit and harm (see "rating values"); and assistance in the final decision making (see "Decision making models").

#### Rating values

**Figure 2:**
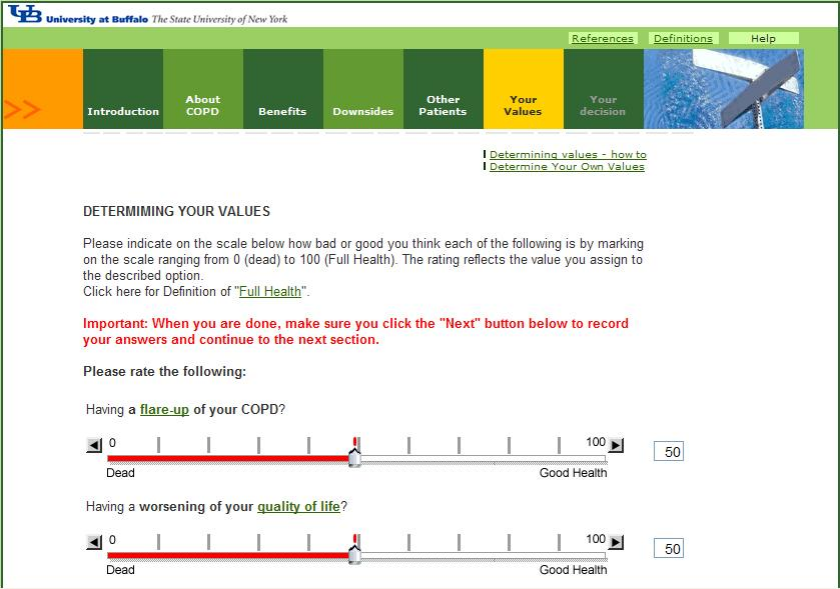
Screenshot showing the value rating instrument.

The DA asks the patient to assign the value she attaches to each of the potential outcomes (benefits and harms) of using inhaled steroids. The value rating instrument is a horizontal feeling-thermometer with values ranging from 0 ("death") to 100 ("Full Health") by increments of 1 unit. The patient moves the cursor of the scale to assign her value for a specific outcome. A box adjacent to the scale indicates the exact value being assigned. The scales for the different outcomes are stacked vertically to enable the patient to compare them visually. (Figure 2)

#### Decision making models

**Figure 3:**
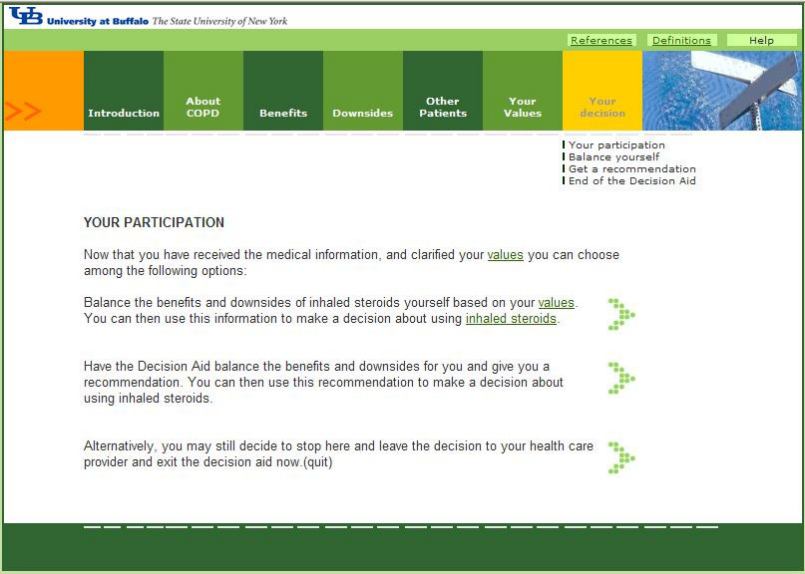
Screenshot showing how the DA provides the user with different decision making models.

The DA, by providing the medical information and clarifying patient values, allows the patient to choose one of four possible decision making models[21,22] Under the "informed decision making model" the patient opts for integrating herself the medical information with her values to make a decision. Under the "physician as an agent model" the patient opts for the DA to integrate the information and the values and provide a recommendation "to use" or "not to use" inhaled steroids. This recommendation is determined by a decision analysis combining outcomes probabilities and the patient assigned values (the expected utility theory). Under the "shared decision making model" the patient reviews the medical information in the DA and goes through the value clarification process, first, and then and makes the decision together with his health care provider. Finally, under the "paternalistic model" the patient can quit the DA at any time and leave the entire decision to her health care provider. (Figure 3)

#### Navigation

The user can access the first page of any section from any page of the DA, and all pages of a particular section from any page of that section. Additional features include hyperlinks to references and definitions of technical words that pop-up in small-size windows. The navigation thus permits access to medical information when needed at any step of the DA.

### Pilot testing of the Decision Aid

Table 1 lists the characteristics of the patients who participated in the pilot testing of the DA. Table 2 lists the results of the pilot testing. Participants provided positive feedback regarding the design, pictures, understandability, user friendliness, perceived required time, explanations, and amount of information in the DA. They felt comfortable and satisfied using it. However they had neutral opinion about the clarity of statistical explanations, the explanation of the concept of values, the helpfulness of the DA and the perceived improvement in knowledge. Both the knowledge and the decisional conflict scales improved significantly after review of the DA. The mean needed time to finish reviewing the DA was 32 min and 75% of patients needed assistance in using the appropriate buttons to navigate the DA.

## Discussion

We have developed a DA for COPD patients considering inhaled steroid therapy. The DA is a computer-based interactive tool structured following the Ottawa Decision Support Framework and integrating four decision-making models (the paternalistic model, the informed decision making model, the physician as an agent model and the shared decision making model). In practice, patients could briefly review the DA with the health care provider at the time of the medical encounter and then complete a detailed review on their own. The most efficient way of completing the decision aid is thus one that does not require presence of a health care provider. COPD patients evaluated positively most of the features of the DA, and showed significant improvement on both the knowledge and the decisional conflict scales.

The DA has several strengths. First, it meets 14 of the 23 quality criteria for "content" and 15 of the 19 quality criteria for "development process" recently proposed by the International Patient Decision Aid Standards (IPDAS) Collaboration [23]. Second, we used a rigorous evidence-based approach to gather and summarize the evidence provided in the decision aid. Third, we have integrated into the decision aid several decision making models to respond to different decision making preferences. We know of no other decision support tool that provides this flexibility, which responds to the variability in decision-making style preferences among patients [24]. Fourth, we assessed patient values using a quantitative method. Fifth, the computer-based format offers the advantages of ease of access, convenience, and ease of update.

The decision aid has some limitations. First, we have not evaluated yet the effectiveness of the DA in terms of the impact on the decision processes or decision quality. While the pilot testing in 8 patients provided us with important information, it is insufficient for claiming its widespread use. Second, the computer-based format of the DA might not be ideal for older patients. *However, the proportion of COPD patients who are computer literate should be on the rise*. Third, the DA does not individualize the risk of developing specific outcomes as in other DAs [25]. We would integrate such function when the necessary statistical prediction models become available.

Other DA developers have also described the challenge of how to communicate risk to patients [24,26]. While the literature suggests that different presentation formats lead to different decisions [27], we do not know which format promotes decisions most consistent with patient values and preferences. An international series of randomized trials (Health Information Project: Presentation Online or HIPPO) are currently underway to try to answer that question [28].

Wilson et al. developed a DA to assist patients with COPD in advance planning for life-threatening exacerbations of their disease [29]. For patients participating in an evaluation study of the decision aid, the burden of treatment (mechanical ventilation in that case) was also an important consideration in making the decision. In addition, about a quarter of patients in that study did not completely comprehend the DA suggesting that this relatively older population might find it challenging to use decision support tools.

This DA may benefit patients with a baseline uncertainty about their choice, similarly to findings in studies of other DAs [30]. Patients who have strong prior preference may still benefit from the medical information the DA provides. The DA will also benefit patients who want to be involved in decision making given patients vary in their preferences for such involvement [31].

## Conclusion

We are planning to conduct a pragmatic clinical trial evaluating the impact of the decision aid on decision processes and decision quality [32]. In that trial we intend to compare different formats of the DA (computer based, paper based) in order to identify the most cost effective option [33]. The DA presents an opportunity to study different ways of presenting information and of eliciting values and evaluate their effect on the decision making process comparatively. It can also serve as a template for the development of other DAs related to emerging COPD treatments that involve a benefit risk tradeoff (e.g. protease inhibitors).

## Disclaimer

The Decision Aid for making decisions about using inhaled steroids in COPD (DA) is not a substitute for medical advice, examination, diagnosis treatment or judgment of a physician or health care professional. If you have any concerns about your health, talk to a doctor. Also, do not disregard or delay seeking medical advice because of something you read on the DA. The information found on the DA is to be used solely for informational purposes. Additionally, in spite of our best efforts, the information in the DA may become out of date over time. The DA team accepts no liability for the accuracy or completeness or use of, nor any liability to update, the information or materials provided in the DA.

## Abbreviations

Decision aid (DA)

Chronic Obstructive Pulmonary Disease (COPD)

Health related quality of life (HRQL)

Ottawa Decision Support Framework (DSF)

International Patient Decision Aid Standards (IPDAS) Collaboration

## Competing interests

The author(s) declare that they have no competing interests.

## Authors' contributions

EAA participated in the acquisition of funding, the design of the study, the development of the DA, data collection, data analysis and drafting of the manuscript. BJBG participated in the acquisition of funding, the development of the DA and data collection. GHG and VMM participated in the design of the study and the development of the DA. HJS conceived of the DA and participated in the acquisition of funding, the design of the study, the development of the DA, data analysis and drafting of the manuscript. All authors critically revised the manuscript for important intellectual content and gave the approval of the version to be published.

## Pre-publication history

The pre-publication history for this paper can be accessed here:



## Supplementary Material

Additional file 1 Inhaled steroids in COPD patients' specific Knowledge scale. Reproduces the Inhaled steroids in COPD patients' specific Knowledge scale Click here for file

Additional file 2 The DA Structure. Describes the structure of the Decision Aid. Click here for file

## Figures and Tables

**Table 1:** Baseline characteristics of pilot testing patients

**Variable**	**Respondents (N= 8)**
	**Mean (SD)**

Age	69 (7.2)
	**n (%)**
Gender (Female)	3 (37.5)
Educational level	
Less than high school	1 (12.5)
high school	5 (62.5)
College	1 (12.5)
graduate degree	1 (12.5)
Ethnic group	
Caucasian	7 (87.5)
African-American	1 (12.5)
Computer user	2 (25.0)
Internet user	1 (12.5)

**Table 2:** Results of the pilot testing

**Variable**	**Value**	**P value**
Design	4.1 (0.6)	0.002
Pictures	4.5 (0.5)	0.000
Understandability	4.4 (0.5)	0.000
User Friendliness	4.5 (0.5)	0.000
Perceived required time	4.6 (0.5)	0.000
Explanation	4.4 (0.5)	0.000
Amount of information	4.8 (0.5)	0.000
Statistical information clarity	3.9 (1.2)	0.111
Concept of values explanation	3.8 (1.0)	0.080
Comfortable using the Aid	4.5 (0.5)	0.000
Decision Aid helpfulness	3.8 (1.3)	0.008
Knowledge improvement	3.9 (1.0)	0.041
Satisfaction	4.3 (0.7)	0.002
Knowledge scale Pre *	4.3 (1.6)	0.008
Knowledge scale Post	6.8 (0.9)	
Decisional Conflict scale Pre §	2.4 (0.7)	0.008
Decisional Conflict scale Post	1.5 (0.4)	
Number of minutes to complete the DA mean (sd)	32.0 (4.5)	N/A
Need for assistance to use the DA n (%)	6 (75%)	

* Min = 0; Max = 10

§ 5 point Likert scale with 1 = low decisional conflict score; 5 = high decisional conflict score.
