# Supplementary material for: A Decision Aid for COPD patients considering inhaled steroid therapy: development and before and after pilot testing
Source: BMC Med Inform Decis Mak. 2007 May 15;7:12. doi: 10.1186/1472-6947-7-12 (PMC1877801; doi:10.1186/1472-6947-7-12)
Supplement: Additional file 1 — Inhaled steroids in COPD patients' specific Knowledge scale. Reproduces the Inhaled steroids in COPD patients' specific Knowledge scale [file 1472-6947-7-12-S1.doc]

**Additional file 1.** Inhaled steroids in COPD patients’ specific Knowledge scale

| Here are some questions about COPD and inhaled steroids. Don’t worry if you can’t remember everything…we did not expect you to memorize the information you received. But it would help us learn what things impressed you enough that you can recall them readily.   1. COPD can be cured   True False Unsure   1. Smoking cessation can slow the progression of COPD   True False Unsure   1. Inhaled steroids are the mainstay of treatment of COPD   True False Unsure   1. Inhaled steroids need to be used over a long period of time (months to years)   True False Unsure   1. Inhaled steroids prolong survival   True False Unsure   1. Inhaled steroids reduce the number of hospital admissions   True False Unsure   1. Inhaled steroids reduce the pace with which quality of life declines   True False Unsure   1. Inhaled steroids increase the chance of bruising   True False Unsure     1. Inhaled steroids increase the chance of getting headaches   True False Unsure   1. Inhaled steroids increase the chance of getting oral thrush   True False Unsure |
| --- |
