# Supplementary material for: A Decision Aid for COPD patients considering inhaled steroid therapy: development and before and after pilot testing
Source: BMC Med Inform Decis Mak. 2007 May 15;7:12. doi: 10.1186/1472-6947-7-12 (PMC1877801; doi:10.1186/1472-6947-7-12)
Supplement: Additional file 2 — The DA Structure. Describes the structure of the Decision Aid. [file 1472-6947-7-12-S2.doc]

**Additional file 2.** The DA Structure.

| **Part I – Medical Information Delivery** | |
| --- | --- |
| **Section** | **Page** |
| **Introduction** | - The purpose of the DA - How a DA works - The different ways the patient can use the DA - The navigation of the DA |
| **About COPD** | - COPD as a disease - Treatment alternatives - Inhaled steroids - Summary of benefits and harms |
| **Benefits** | - Decreased incidence of exacerbations - Slower decline of Health Related Quality of Life |
| **Harms** | - Ecchymosis - Oral candidiasis - Throat irritation - Burden of using inhaled steroids |

| **Part II – Decision Making Process** | |
| --- | --- |
| **Section** | **Page** |
| **Other patients** | - Introduction to the hypothetical case scenarios - Mr. Smith case scenario - Mrs. Jackson case scenario - Mrs. Brown case scenario |
| **Your values** | - Explanation on values measurement - Values measurement |
| **Your decision** | - Different decision making models - Decision making based on the chosen model - End of the DA |

In addition to these pages, the DA includes a glossary page and a references page
